# Perinatal depressive symptoms and received support from health professionals: results from the national FinChildren survey

**DOI:** 10.1080/02813432.2025.2546428

**Published:** 2025-08-19

**Authors:** Heidi Kesanto-Jokipolvi, Olli Kiviruusu, Maaret Vuorenmaa, Eetu Ervasti, Reija Klemetti

**Affiliations:** ^a^ Unit of Health Sciences, Faculty of Social Sciences, Tampere University, Tampere, Finland; ^b^ Promotional and Preventive Work Unit, Department of Healthcare and Social Welfare, Finnish Institute for Health and Welfare, Helsinki, Finland; ^c^ Welfare Epidemiology and Monitoring Unit, Department of Public Health, Finnish Institute for Health and Welfare, Helsinki, Finland; ^d^ Faculty of Medicine, Department of Public Health, University of Helsinki, Helsinki, Finland

**Keywords:** Mental health, parents, depression, prenatal, postpartum, support, health professionals

## Abstract

**Background:**

Perinatal depressive symptoms affect one in 10 parents. However, there is a lack of knowledge on issues related to early and appropriate support for depressive parents. The study investigated what kind of support depressive parents need and receive prenatally from health professionals and whether the received support moderates the association between prenatal depressive symptoms and postpartum mental health.

**Methods:**

The FinChildren survey for parents of babies aged 3–6 months (8977 mothers, 5825 fathers) was conducted in 2020. Parents evaluated their current mental health at the time and prenatal depressive symptoms and support needed (e.g. parenthood, mood, fear of childbirth) retrospectively.

**Results:**

Prenatal depressive symptoms (mothers 29.0%, fathers 12.7%) were associated with the need for all types of support, and inadequate support was associated with poorer postpartum mental health for all parents. For prenatal depressive parents, prenatal support for mood, and for prenatally depressive fathers, support in the case of fear of childbirth were important elements in reducing postpartum depressive symptoms or mental strain.

**Limitations:**

The study design was retrospective and cross-sectional. A screening tool was used to identify prenatal depressive symptoms without a clinically relevant cut-off point.

**Conclusions:**

Prenatally depressive parents’ support needs do not only concern mental health. Unmet support needs during pregnancy were highly predictive for postpartum depressive symptoms or mental strain. Adequate support for prenatal mood and in the case of paternal fear of childbirth is important, but further research is needed on the most relevant combinations of support issues and practices to support depressive parents.

## Introduction

The transition into parenthood is associated with the potential for mental health disorders [1,2]; the parental risk of mental health disorders increases, especially during the first year after the birth of the child [3]. In fact, prenatal and postpartum depression are common, and one in ten parents suffers from depression during the perinatal period [4–6]. There is tentative evidence to suggest that early interventions during pregnancy and after childbirth are cost-effective at tackling parental mental health problems [7]. Primary healthcare has performed an essential role in identifying parental depression [8,9]. However, an imbalance between the need for support and the support received by depressive parents has also been observed recently [10]. Considering that psychological interventions and psychoeducation by non-specialist nurses and midwives have successfully reduced maternal depressive symptoms [11,12], primary health care needs to play a new role in providing early treatment as well (see [13]).

Focusing attention on mental health in pregnancy is essential because perinatal depressive symptoms can be chronic [14,15], and prenatal depression is a strong predictor of postpartum depression in parents [14,16–18]. Parental depression is an important field of research, not only because of the harmful effects on the parents themselves, but because parental depression is associated with low satisfaction with family life [19], low satisfaction with the marital relationship [20], an increased risk of depressive symptoms in spouses [14,20,21], and an increased risk of internal and external emotional problems, such as anxiety, in children in the long and short term [22–25]. Prenatal depression and anxiety also increase the risk of non-physiological birth [26], such as a caesarean section, which is in turn associated with many complications for the mother and future pregnancies, as well as long-term outcomes in childhood [27]. Negative experiences in childbirth are also known to be a risk factor for postpartum depression [28].

Recent studies have reported encouraging results related to interventions that provide parenting support to depressive parents, for example [29]. So, it seems that support for parental depression should not only target parental mental health; parents may also need support with other issues, such as life management, preparing for parenthood, partnership, and other open-ended issues, which are poorly known. Thus, more knowledge is needed on the kinds of support parents need and what approaches are useful for reducing parental depressive symptoms. Knowledge of suitable treatments and support for paternal depressive symptoms is also scant [7,30,31].

The present study retrospectively examines the prenatal period and its association with parental postpartum mental health. The study questions are: (1) How common are prenatal and postpartum depressive symptoms and mental strain among Finnish mothers and fathers with babies aged 3–6 months? (2) Are parental prenatal depressive symptoms associated with support needed from health professionals during pregnancy? (3) Does inadequate support during pregnancy predict postpartum depressive symptoms and mental strain? (4) Does support received from health professionals during pregnancy moderate the association between parental prenatal depressive symptoms and postpartum depressive symptoms and mental strain?

## Methods

### Context of the study

National legislation in Finland obliges all well-being services counties to organise uniform maternity and child health clinic services. Maternity and child health clinic services include regular health examinations and healthcare counselling during pregnancy and after childbirth. Healthcare counselling is evidence-based and considers the families’ individual needs. Counselling is provided for both parents in families who are expecting the baby [32,33]. Maternity clinic services include at least eight health examinations during pregnancy and two health examinations after childbirth. Families who are expecting their first baby are recommended to receive one home visit and one extra health examination during pregnancy [34]. For children, 15 visits are recommended before the age of six years [33]. Maternity and child health clinic services are free of charge [32] and voluntary for families. In Finland, maternity and child health clinic services reach 99.7% of the pregnant and 99.5% of families whose children are 0–6 years old [35].

### The FinChildren study and participants

The aim of the FinChildren study is to provide real-time information about the well-being and service use of Finnish families with young children for welfare and health promotion at the national level. The database consists of regular nationwide survey data collected from Finnish families and individual-level register data on the health and well-being of children living in Finland.

Data from the FinChildren 2020 survey that concerned both parents (mothers giving birth and other parents) of babies aged 3–6 months were used. The parents’ contact details were obtained from the Digital and Population Data Services Agency’s Population Information System. The sampling criteria were defined as the data of the guardians of all babies born during the examined periods with a permanent address. The Finnish Institute for Health and Welfare invited the parents (mothers *n* = 17,964, other parents *n* = 16,112) by mail, but the parents could fill in a paper questionnaire or respond online between 12th March 2020 and 12th January 2021. The questionnaire was available in Finnish, Swedish, English, Russian, Somali, Arabic, and Northern Sámi (read more about the study protocol in [36]).

### Measures

Parental age and the country of origin (birth country) were delivered by Digital and Population Data Services Agency’s Population Information System. Parental age and the countries of origin (birth country) were analysed as categorical variables. Age was categorised <30 years and ≥30 years, and the countries of origin were divided into Finland and other. Other sociodemographic background variables were derived from the parental questionnaires (education, financial situation in the family, and number of children). The parents’ level of education (‘what is your level of education?’) was measured on a 9-point-scale (have not finished comprehensive school, comprehensive school, occupational course or on-the-job training, matriculation examination (upper secondary school), vocational qualification or specialist vocational qualification from a vocational institution, lower degree from a university of applied sciences or university (bachelor’s degree), higher university of applied sciences or university degree (master’s, licentiate, or doctoral degree), cannot say and were classified into two categories for the analysis: high (at least a bachelor’s degree) and lower education (other options). The financial situation was self-rated (how would you rate your family’s financial situation?) which was construed and analysed as a three-category variable: ‘low’ including very poor and fairly poor, ‘moderate’, and ‘high’ including fairly good and very good. The survey included a question regarding previous children: ‘In addition to the baby, are there other children aged under 18 living with your family (including children living in your family part time)?’ The response options, used dichotomously in the analysis, were ‘yes = 1’ or ‘no = 0’.

#### Retrospective measures regarding the prenatal period

Parental prenatal depressive symptoms were asked of both parents when their baby was 3–6 months old using the following screening tool question: ‘During the pregnancy, did you have at least one consecutive period of two weeks when you felt particularly worried, unhappy, or depressed?’ The respondents answered using the options ‘yes’ or ‘no’, which were the outcome categories in the analyses.

The parental need for support from professionals and support received from professionals in the prenatal period were enquired: ‘Did you receive adequate support from various professionals (including the maternity clinic) during the pregnancy for the following issues?’ The issues asked included parenthood, mood swings (only from mothers), mood (only from fathers), depression (only for mothers), personal coping, intimate relationship, fear of childbirth, and preparing for labour and birth. The response alternatives were: (1) ‘I did not need support’, (2) ‘I received adequate support’, (3) ‘I received support, but it was not adequate’, (4) ‘I would have needed support but did not get it’, and (5) ‘I would have needed support, but I did not bring it up’.

In the analyses, two classifications for parental support needed and parental support received were used. To investigate whether parental prenatal depressive symptoms are associated with support being needed from health professionals during pregnancy, all parents were classified into dichotomic categories: parents who needed support from professionals and parents who did not need support. The moderating effects of support being received from health professionals during pregnancy were examined more closely by dividing the support received into three categories: (1) received adequate support (incl. ‘I received adequate support’), (2) received inadequate or no support (incl. ‘I received support but it was not adequate’, ‘I would have needed support but did not get it’), and (3) the reference group – no need for support (incl. ‘Did not need support’, ‘I would have needed support but I did not bring it up’).

From the point of view of health professionals, appointments are customised by parents implicitly expressed needs. If parents do not express support needs, it is more difficult for health professionals to provide adequate support. Therefore, parents who did not express a need for support were systematically included in the group ‘Did not need support’, although this does not mean that they do not actually need support.

#### Measures of current (postpartum) mental health

Maternal postpartum depressive symptoms were measured using the 10-item version (CES-D-10) [37] of the Center for Epidemiologic Studies Depression Scale [38], which describes symptoms of depression in the past month. The items in these 10 questions were: (1) felt depressed, (2) felt that everything required an effort, (3) sleep was restless, (4) felt happy, (5) felt lonely, (6) found that people were unfriendly, (7) enjoyed life, (8) felt sad, (9) felt that others disliked them, and 10) found it difficult to get going. The response alternatives were: (1) seldom or never (0 points), (2) sometimes (1 point), (3) often (2 points), and (3) all of the time (3 points). The scores for items 4 and 7 are reversed, after which the item scores were summed (range 0–30). The respondents were treated as two groups in the analysis: 1 = symptoms of depression, i.e. the total sum score ≥10 or more [37], 0 = the total sum score <10.

The mental strain of fathers in the postpartum period was defined using the Mental Health Inventory (MHI-5 scale [39]). Respondents were asked how much of the time over the past four weeks they: (1) felt very nervous, (2) had such a low mood that nothing could cheer them up, (3) felt calm and peaceful, (4) felt downhearted and sad, and (5) had been happy. The response alternatives were: (1) all the time, (2) most of the time, (3) a good bit of the time, (4) some of the time, (5) a little of the time, and (6) none of the time. The scores for items 3 and 5 are reversed, after which the item scores are summed (range 5–30) and converted to a 0–100 scale. Respondents were considered significantly mentally strained if the total score of the responses was ≤52 following the Finnish population studies [40]. A dichotomised ­variable was used in the analysis 1 = total score ≤52, 0 = total score >53.

### Statistical analysis

The prevalence of parental prenatal depressive symptoms and postpartum depressive symptoms were reported in relative proportions. In the first phase, cross-tabulation and the chi-square test between parental prenatal depressive status and sociodemographic factors were calculated to find control and background variables for the prenatal depressive symptoms.

All issues of received support from health professionals were analysed separately. The associations between prenatal depressive symptoms and parental support needs from health professionals during pregnancy were estimated in relative proportions and using logistic regression modelling. Prenatal depressive symptoms were the outcome and parental support needed from health professionals was as an independent variable in the logistic regression models.

To investigate whether inadequate support during pregnancy was associated with postpartum depressive symptoms, the received support from health professionals was first entered as an independent variable into a logistic regression analysis using postpartum depressive symptoms as the outcome (Model 1). In Model 2, the association between received support from health professionals and postpartum depressive symptoms adjusted for prenatal depressive symptoms by entering received support from health professionals and prenatal depressive symptoms at the same time as independent variables in the logistic regression analysis. Finally, an interaction term between the received support from health professionals and prenatal depressive symptoms was added in Model 2 to examine whether the received support moderated the association between prenatal depressive symptoms and maternal postpartum depressive symptoms or paternal mental strain.

All models were adjusted for the parents’ sociodemographic background factors (parents’ education, parental age, financial situation in the family, number of children, and country of origin). Cases with missing values were omitted from the analyses. In the analyses, *p*-values <0.05 were considered statistically significant. Because our primary focus was on estimating effect sizes with confidence intervals rather than on formal hypothesis testing, a correction strategy for multiple comparisons was not applied.

The data were analysed using R [41], version 4.3.0 and SPSS statistical software for Windows, ­version 29.0.

## Results

In total, 8977 parents who gave birth (response rate 50%) and 5843 other parents (response rate 36%) responded. The parents who responded to the survey were slightly older and more educated than those who did not. The response rate also varied by region, and foreign-born parents were underrepresented among the respondents (the exact numbers are available in [36]).

The study population consisted of parents who had answered the question about prenatal depressive symptoms (mothers *n* = 8914; fathers *n* = 5727) and parents who had completed the postpartum mental health indicators of depressive symptoms and mental strain (mothers *n* = 8445; fathers *n* = 5635). Other female parents (*n* = 18) were excluded from the study because this group was very small and could not be treated separately as they deserved. The numbers for the missing data regarding the background variables studied are available in Supplement 1 and for the issues of support from health professionals in Table 1.

About half of the families included in the sample had had their baby before the COVID-19 outbreak, while the rest had their baby during the pandemic. There were no differences in postpartum depression and mental distress between parents who had their baby before and during COVID-19. Mothers who had their babies during the pandemic reported slightly more prenatal depressive symptoms.

### The prevalence of prenatal depressive symptoms and postpartum depressive symptoms and mental strain

In total, the prevalence of prenatal depressive symptoms was 29.0% for mothers and 12.7% for fathers. Postpartum depressive symptoms were found among 14.7% of mothers and postpartum mental strain among 3.9% of fathers.

### The background characteristics of prenatal depressive symptoms

The cross-tabulation and chi-square test of parental prenatal depressive symptoms and socio-demographic factors revealed that lower maternal education, high paternal education, a poorer financial situation in the family, and the father’s country of origin being other than Finland were associated with prenatal depressive symptoms (Supplement 1).

### Prenatal depressive symptoms and support needed from health professionals

The most common issues of support that all parents needed were preparing for labour and birth, parenthood, and personal coping (Table 1). Parents with prenatal depressive symptoms needed health professional support more than other parents across all studied issues. The ORs varied by support issue from 1.16 to 3.75 among prenatally depressive mothers, and from 1.21 to 2.32 among prenatally depressive fathers (Table 2; see also Table 1). Prenatal depressive symptoms had the strongest associations with support needed for depression (OR 3.75) and mood swings (OR 2.96) among mothers, and with support needed for mood (2.32), personal coping (OR 1.92), and fear of childbirth (OR 1.86) among fathers (Table 2).

**Table 1.** Needed health professional provided support during pregnancy among prenatal depressive and other parents.

Health professional provided support	Mothers				Fathers			
	Prenatal depressive symptoms (*n* = 2583)		Other (*n* = 6331)		Prenatal depressive symptoms (*n* = 724)		Other (*n* = 5003)	
	% (*n*)	Missing % (*n*)	% (*n*)	Missing % (*n*)	% (*n*)	Missing % (*n*)	% (*n*)	Missing % (*n*)
Parenthood	69.4 (1792)	1.0 (27)	64.9 (4108)	1.2 (79)	61.3 (444)	2.3 (17)	51.4 (2574)	2.3 (114)
Personal coping	73.2 (1890)	1.1 (29)	51.5 (3258)	1.2 (75)	51.2 (371)	3.5 (25)	27.8 (1390)	2.7 (134)
Intimate relationship	40.8 (1054)	0.9 (24)	29.6 (1875)	1.0 (61)	44.5 (322)	3.0 (22)	29.4 (1469)	2.6 (130)
Fear of childbirth	49.3 (1273)	0.8 (21)	33.2 (2105)	1.1 (68)	28.3 (205)	3.0 (22)	15.6 (782)	2.7 (133)
Preparing for labour and birth	80.1 (2069)	0.8 (20)	77.2 (4890)	1.0 (64)	50.6 (366)	3.3 (24)	45.2 (2263)	2.7 (134)
Mood swings (only for mothers)	63.7 (1645)	1.0 (25)	32.9 (2083)	1.0 (62)	NA		NA	
Depression (only for mothers)	39.3 (1014)	1.2 (30)	12.4 (787)	1.0 (66)	NA		NA	
Mood (only for fathers)	NA		NA		50.7 (367)	2.8 (20)	23.5 (1174)	2.5 (125)

NA: not available.

**Table 2.** The associations between parental needed support from health professionals and prenatal depressive symptoms (or, 95% CI).^a^

Health professionals provided support	Mothers			Fathers		
	OR^b^	95% CI		OR^b^	95% CI	
Parenthood	**1.16**	1.05	1.29	**1.41**	1.18	1.69
Personal coping	**1.97**	1.79	2.17	**1.92**	1.62	2.28
Intimate relationship	**1.35**	1.22	1.5	**1.36**	1.14	1.62
Fear of childbirth	**1.93**	1.76	2.13	**1.89**	1.55	2.29
Preparing for labour and birth	**1.17**	1.05	1.32	**1.21**	1.01	1.44
Mood swings (only for mothers)	**2.96**	2.69	3.27	NA	NA	NA
Depression (only for mothers)	**3.75**	3.34	4.21	NA	NA	NA
Mood (only for fathers)	NA	NA	NA	**2.32**	1.95	2.76

NA: not available.

Statistically significant results are indicated in bold font.

^a^ Adjusted by parental age, education, financial situation, country of birth, and the number of children in the family.

^b^ Reference groups: prenatally non-depressed mothers.

### Prenatal depressive symptoms and prenatal support predicting parental postpartum depression and mental strain

In Table 3, Model 1 showed that prenatal depressive symptoms as well as inadequate or no support from health professionals during pregnancy were associated with higher levels of maternal postpartum depressive symptoms and paternal mental strain. The models also indicated that receiving adequate support from health professionals during pregnancy was associated with higher levels of postpartum depressive symptoms among mothers, but not with paternal mental strain.

**Table 3.** The associations between parental postpartum depression and mental strain and support provided by the health professionals during pregnancy (or, CI 95%).^a^

	Model 1: ORs,^b^ maternal postpartum depression and paternal mental strain by provided support						Model 2: Adjusted ORs,^c^ maternal postpartum depression and paternal mental strain by provided support					
	Mothers			Fathers			Mothers			Fathers		
	OR	95% CI		OR	95% IC		OR	95% CI		OR	95% CI	
Prenatal depressive symptoms	5.06	4.44	5.76	7.52	5.62	10.1						
**Health professionals provided support**												
Parenthood												
Inadequate or no support	**3.94**	3.14	4.94	**2.93**	1.8	4.65	**3.18**	2.5	4.05	**2.08**	1.24	3.4
Adequate support	1.05	0.91	1.21	0.97	0.7	1.33	1.01	0.87	1.16	0.84	0.6	1.17
No (or no expressed) need for support	1			1			1			1		
Personal coping												
Inadequate or no support	**6.43**	5.3	7.79	**5.91**	3.9	8.8	**4.37**	3.57	5.36	**3.57**	2.29	5.47
Adequate support	**1.49**	1.29	1.71	1.11	0.77	1.58	**1.28**	1.11	1.49	0.97	0.67	1.39
No (or no expressed) need for support	1			1			1			1		
Intimate relationship												
Inadequate or no support	**5.32**	4.16	6.79	**4.54**	2.95	6.83	**4.31**	3.32	5.6	**3.09**	1.95	4.80
Adequate support	**1.18**	1.02	1.37	0.84	0.56	1.22	1.10	0.95	1.28	0.81	0.54	1.19
No (or no expressed) need for support	1			1			1			1		
Fear of childbirth												
Inadequate or no support	**3.93**	3.24	4.76	**2.84**	1.43	5.17	**2.92**	2.38	3.57	1.71	0.84	3.22
Adequate support	**1.48**	1.29	1.7	0.95	0.6	1.43	**1.25**	1.08	1.44	0.77	0.48	1.18
No (or no expressed) need for support	1			1			1			1		
Preparing for labour and birth												
Inadequate or no support	**2.15**	1.79	2.6	**1.77**	1.06	2.84	**1.81**	1.48	2.2	1.30	0.76	2.15
Adequate support	0.93	0.79	1.09	0.67	0.47	0.94	0.90	0.76	1.07	**0.64**	0.44	0.90
No (or no expressed) need for support	1			1			1			1		
Mood swings (only from mothers)												
Inadequate or no support	**8.08**	6.54	9.97	NA	NA	NA	**5.06**	4.04	6.32	NA	NA	NA
Adequate support	**1.82**	1.58	2.08	NA	NA	NA	**1.36**	1.17	1.57	NA	NA	NA
No (or no expressed) need for support	1						1					
Depression (only from mothers)												
Inadequate or no support	**12.42**	9.56	16.21	NA	NA	NA	**7.02**	5.33	9.28	NA	NA	NA
Adequate support	**2.49**	2.13	2.9	NA	NA	NA	**1.77**	1.5	2.08	NA	NA	NA
No (or no expressed) need for support	1						1					
Mood (only from fathers)												
Inadequate or no support	NA	NA	NA	**6.17**	4.01	9.31	NA	NA	NA	**3.36**	2.12	5.22
Adequate support	NA	NA	NA	1.24	0.85	1.77	NA	NA	NA	1.02	0.70	1.48
No (or no expressed) need for support				1								

NA: not available.

Statistically significant results are indicated in bold font.

^a^ All models are adjusted by parental age, education, financial situation, country of birth, and the number of children in the family.

^b^ Univariate model for each support issue provided by health professionals.

^c^ The model for each support issue adjusted by status of prenatal depressive symptoms.

The above associations mainly remained in Model 2, which adjusted for prenatal depressive symptoms, but with the following exceptions: adequate support for the intimate relationship did not reach statistical significance among mothers, fathers received adequate support in preparation for labour and birth became statistically significant, while inadequate support for labour and birth lost its statistical significance.

Further analysis confirmed that the associations persisted, even when therapy received and medication use were added to Model 2 (available in Supplement 2).

### Moderating role of received support

The interaction terms between prenatal depressive symptoms and inadequate or no support did not achieve statistical significance, indicating that the association between inadequate or no support and postpartum depressive symptoms or mental strain was similar among prenatally depressive parents and the other parents. However, three statistically significant interactions between prenatal depressive symptoms and adequate parental support from health professionals were found: maternal mood swings (OR 0.66, 95% CI 0.50–0.88), paternal mood (OR 0.43, 95% CI 0.20–0.90), and paternal fear of childbirth (OR 0.29, 95% CI 0.12–0.69).

Adequate support for mood swings among prenatal depressive mothers decreased the OR (1.1, 95% CI 0.91–1.34) for postpartum depressive symptoms to the control group level, while for the other mothers the OR was still 1.70 (95% CI 1.37–2.09). Paternal ORs for postpartum mental distress were lower among prenatally depressive fathers when they had received support for mood (OR 0.41, 95% CI 0.2–0.77) or fear of childbirth (OR 0.63, 95% CI 0.35–1.08) compared to the corresponding ORs (1.43, 95% CI 0.79–2.45 and 1.56, 95% CI 0.94–2.51, respectively) for the other fathers.

## Discussion

The results showed that parental prenatal depressive symptoms are common in the Finnish population and predicted maternal postpartum depressive symptoms and paternal mental strain. We found that parental prenatal depressive symptoms are associated with support needs for many issues, not only mental health. Meanwhile, inadequate or no support from health professionals during pregnancy was associated with the higher prevalence of maternal depressive symptoms and paternal mental distress for all parents in the postpartum period. For prenatal depressive mothers and fathers, prenatal support for mood, and for prenatally depressive fathers, support in the case of fear of childbirth were important elements in reducing postpartum depressive symptoms/mental strain.

More than one in four mothers and more than one in ten fathers reported prenatal depressive symptoms in the study. This is a higher prevalence of parental depressive symptoms than in the previous studies internationally [4,6] or in the Finnish population [14], which is likely due to the measure used. Sensitivity and specificity in single-question screening tools for prenatal depressive symptoms are compromised [42]. However, the finding that these symptoms were predictive for postpartum depressive symptoms and paternal mental strain is consistent with the literature [14,16–18].

The study showed that parents with prenatal depressive symptoms reported needing more support from health professionals compared to other parents, but inadequate or no support from health professionals during pregnancy was associated with a higher prevalence of postpartum depressive symptoms and mental strain among all parents. Prior studies have found that a lack of social support is a risk factor for perinatal depressive symptoms [16,43,44], which may explain the importance of support from health professionals. The finding that adequate support was also associated with higher levels of maternal postpartum depression does not mean that adequate support is useless. Rather, the result reflects that the need for support might be the first sign of mental health vulnerability, and cases of depression can be prevented with adequate support and within a relatively short time frame during the perinatal phase.

In this study, prenatally depressive parents who received adequate support for their mood problems from health professionals during pregnancy reported less postpartum depressive symptoms and mental distress compared to the control group. This is supported by previous evidence; psychological interventions and psychoeducation by non-specialist nurses and midwives are beneficial in reducing perinatal depressive symptoms and anxiety [11,12], and psychological interventions in the prenatal period may reduce both prenatal and postnatal depression symptoms [45].

We also found that prenatally depressive fathers had less postpartum mental distress if they had received adequate support in the case of fear of childbirth. This result was not detected for mothers. Adequate support might be expected to mitigate postpartum mental health problems regardless of parental role, as depressive symptoms and poorer mental health are known to be associated with the fear of childbirth for both parents [46,47]. Direct comparisons between parents are difficult, however, because different measures of parental postpartum mental health were used. On the other hand, the analysis indicated that fear of childbirth was more common among fathers with prenatal depressive symptoms than among other fathers, while among mothers, fear of childbirth was more evenly distributed (see Table 1), which may partly explain the differing results between the parents. Childbirth, as a concrete event, may also play a more crucial role for fathers in the transition to parenthood, and the adequate support in the case fear of childbirth may facilitate this transition, especially for vulnerable fathers.

This study identified various support needs among prenatally depressive parents. There are few studies examining prenatally depressive parents’ perceived support needs from health professionals, but mothers’ satisfaction with emotional support provided by health professionals in the prenatal period has been associated with lower postpartum stress levels [48]. The results of a Finnish study based on interviews with public health nurses indicate that interactions with mothers at risk of postnatal depression should include discussions and counselling and most importantly be holistic and co-operative with the whole family unit [49]. Nevertheless, further research is needed to find which practices or combinations of support issues are most useful in reducing parental depressive symptoms.

In the future, efforts to develop perinatal mental healthcare can be expected to produce a good return on investments [7,50]. The strengthening of the primary care role in parental depression care supports the implementation of new evidence-based recommendations for perinatal mental healthcare [51] and may enhance fathers’ access to treatment. Regular health examinations in maternity clinics would create good opportunities to provide more low-threshold mental health support for all parents. Adequate and universally provided support from health professionals benefits parents with and without prenatal depressive symptoms. However, more knowledge is needed about relevant practices to support parents in the prenatal period.

### Limitations

Our study was cross-sectional and addressed the prenatal phase retrospectively. Retrospective study designs are prone to recall bias, and depressive symptoms themselves may influence how parents evaluate received support from health professionals. Therefore, we cannot know how much or how little support the parents received from health professionals during pregnancy, nor can we draw conclusions about potential causal mechanisms. In addition, the results should be treated and interpreted with caution because a single question was used for prenatal depressive symptoms screening, not a clinically relevant cut-off point. Secondly, the study population is not a fully representative sample of Finnish parents. A longitudinal study and more clinically relevant measures of parental perinatal mental health are needed to confirm our results.

## Conclusion

The study contributes to the existing literature by showing that prenatal depressive parents’ support needs do not only concern mental health, and unmet support needs were highly predictive of postpartum depressive symptoms and mental strain among all parents. Therefore, universally provided support from health professionals benefits parents with and without prenatal depressive symptoms. Adequate support for mood and in the case of the paternal fear of childbirth seems to be most promising for prenatally depressive parents, but further study is needed to find which practices or combinations of support issues are most relevant in reducing parental depressive symptoms.

## Supplementary Material

Supplemental Material

## Data Availability

The dataset in this article is not publicly available. The original dataset is available upon reasonable request (info(at)findata.fi).
