# Supplementary material for: Perinatal depressive symptoms and received support from health professionals: results from the national FinChildren survey
Source: Scand J Prim Health Care. 2025 Aug 19;44(1):1–11. doi: 10.1080/02813432.2025.2546428 (PMC12918359; doi:10.1080/02813432.2025.2546428)
Supplement: Supplemental Material [file IPRI_A_2546428_SM6974.docx]

**Supplement 1**

**Background characteristics of mothers and fathers by prenatal depressive symptoms and in total with the number of missing values.**

|  | **Mothers** | | | | **Fathers** | | | |
| --- | --- | --- | --- | --- | --- | --- | --- | --- |
| Characteristic | Prenatal depressive symptoms % (n=2583) | Other % (n=6331) | p^1^ | Total % (n=8914) | Prenatal depressive symptoms % (n=724) | Other % (n=5003) | p^1^ | Total % (n=5745) |
|  |  |  |  |  |  |  |  |  |
| **Age, mean (SD)** | 31.5 (5.2) | 31.7 (5.1) |  | 31.6 (5.1) | 34.3 (6) | 33.8 (5.8) |  | 33.9 (5.8) |
| **Age group** |  |  | 0.107 | missing: none |  |  | 0.469 | missing=none |
| 30+ | 28.4 (1677) | 71.6 (4223) |  | 100 (5900) | 12.9 (579) | 87.1 (3911) |  | 100 (4490) |
| < 30 | 30.1 (906) | 69.9 (2108) |  | 100 (3014) | 12 (151) | 88 (1104) |  | 100 (1101) |
| **Education** |  |  | **0.004** | missing n=36 (0.4%) |  |  | **<0.001** | missing n=23 (0.4 %) |
| High | 27.9 (1497) | 72.1 (3874) |  | 100 (5371) | 14.4 (422) | 85.6 (2509) |  | 100 (2931) |
| Lower | 30.7 (1077) | 69.3 (2432) |  | 100 (3509) | 10.9 (304) | 89.1 (2493) |  | 100 (2797) |
| **Financial situation** |  |  | **<0.001** | missing n=62 (0.7%) |  |  | **<0.001** | missing n=77 (1.3%) |
| High | 25.3 (1460) | 74.7 (4315) |  | 100 (5775) | 10.4 (399) | 89.6 (3453) |  | 100 (3852) |
| Moderate | 33.8 (892) | 66.2 (1744) |  | 100 (2636) | 27.5 (65) | 72.5 (171) |  | 100 (236) |
| Low | 47 (208) | 53 (235) |  | 100 (443) | 16.2 (256) | 83.8 (1328) |  | 100 (1584) |
| **Previous children** |  |  | 0.442 | missing n=40 (0.4%) |  |  | 0.191 | missing n=49 (0.8%) |
| Yes | 29.3 (1394) | 70.7 (3357) |  | 100 (4751) | 13.2 (385) | 86.8 (2527) |  | 100 (2912) |
| No | 28.6 (1180) | 71.4 (2946) |  | 100 (4126) | 12.1 (340) | 87.9 (2465) |  | 100 (2805) |
| **Country of origin** |  |  | 0.783 | missing=none |  |  | **<0.001** | missing=none |
| Finland | 29 (2303) | 71 (5632) |  | 100 (7935) | 11.5 (574) | 88.5 (4418) |  | 100 (4992) |
| Other | 28.6 (280) | 71.4 (699) |  | 100 (979) | 20.7 (156) | 79.3 (597) |  | 100 (753) |
| ^1^Chi-square test  Statistically significant is in bold | | |  |  |  |  |  |  |

**Supplement 2.**

**The associations between parental postpartum depression and mental strain and support provided by the health professionals during pregnancy (OR, CI 95 %), adjusted for the background factors,^1^ prenatal depressive symptoms, and the medical treatments.**

|  | Mothers | | | Fathers | | |
| --- | --- | --- | --- | --- | --- | --- |
|  | OR | 95 % CI | | OR | 95 % CI | |
| **Health professionals provided support** |  |  |  |  |  |  |
|  |  |  |  |  |  |  |
| **Parenthood** |  |  |  |  |  |  |
| inadequate or no support | **3.17** | 2.47 | 4.05 | **1.74** | 1.01 | 2.90 |
| adequate support | 0.99 | 0.85 | 1.15 | 0.76 | 0.54 | 1.07 |
| no need (or expressed) for support | 1 |  |  | 1 |  |  |
| **Personal coping** |  |  |  |  |  |  |
| inadequate or no support | **4.23** | 3.43 | 5.20 | **3.28** | 2.06 | 5.10 |
| adequate support | **1.25** | 1.07 | 1.45 | 0.86 | 0.58 | 1.26 |
| no (or no expressed) need for support | 1 |  |  | 1 |  |  |
| **Intimate relationship** |  |  |  |  |  |  |
| inadequate or no support | **4.25** | 3.25 | 5.56 | **3.02** | 1.87 | 4.74 |
| adequate support | 1.07 | 0.91 | 1.25 | 0.77 | 0.51 | 1.14 |
| no (or no expressed) need for support | 1 |  |  | 1 |  |  |
| **Fear of childbirth** |  |  |  |  |  |  |
| inadequate or no support | **2.86** | 2.32 | 3.52 | 1.63 | 0.78 | 3.12 |
| adequate support | **1.23** | 1.06 | 1.42 | 0.73 | 0.45 | 1.13 |
| no (or no expressed) need for support | 1 |  |  | 1 |  |  |
| **Preparing for labour and birth** |  |  |  |  |  |  |
| inadequate or no support | **1.79** | 1.46 | 2.19 | 1.18 | 0.67 | 1.98 |
| adequate support | 0.88 | 0.74 | 1.04 | **0.56** | 0.38 | 0.80 |
| no (or no expressed) need for support | 1 |  |  | 1 |  |  |
| **Mood swings (only from mothers)** |  |  |  |  |  |  |
| inadequate or no support | **5.05** | 4.01 | 6.35 | NA | NA | NA |
| adequate support | **1.35** | 1.16 | 1.56 | NA | NA | NA |
| no (or no expressed) need for support | 1 |  |  |  |  |  |
| **Depression (only from mothers)** |  |  |  |  |  |  |
| inadequate or no support | **6.92** | 5.20 | 9.25 | NA | NA | NA |
| adequate support | **1.71** | 1.44 | 2.03 | NA | NA | NA |
| no (or no expressed) need for support | 1 |  |  |  |  |  |
| **Mood (only from fathers)** |  |  |  |  |  |  |
| inadequate or no support | NA | NA | NA | **3.12** | 1.93 | 4.94 |
| adequate support | NA | NA | NA | 0.90 | 0.60 | 1.32 |
| no (or no expressed) need for support |  |  |  | 1 |  |  |
| ^1^ All models are adjusted by parental age, education, financial situation, country of birth, and the number of children in the family | | | | | |  |
| Statistically significant results are indicated in bold font |  |  |  |  |  |  |

**Details about the analysis:** All issues of support received from health professionals were analysed separately, as in the main analysis. Outcome: postpartum depressive symptoms (mothers), postpartum mental strain (fathers). Independent variables: support received from health professionals, prenatal depressive symptoms, received medication or therapy (yes=1 or no=0) + background variables (parental age, education, financial situation, country of birth, and the number of children in the family).

Received medical treatment or therapy was mapped by the following question: “Did you tell a professional if you had at least one consecutive period of two weeks when you felt particularly worried, unhappy, or depressed (for example, a nurse, public health nurse, doctor, psychologist, therapist, social worker)? The response options were “yes” or “no”. If the respondent selected “yes”, the following question appeared “What happened next? I received: counselling (yes/no), therapy(yes/no), a diagnosis (yes/no), medication (yes/no)”.
